# Long term results in the life quality of children with obstructive sleep disorders

**DOI:** 10.1016/S1808-8694(15)31382-3

**Published:** 2015-10-17

**Authors:** José Mário de Lima Júnior, Viviane Carvalho da Silva, Marcos Rabelo de Freitas

**Affiliations:** 1 Medical doctor, otorhinolaryngology resident, Otorhinolaryngology Unit, Hospital Universitário Walter Cantídio HUWC./Universidade Federal do Ceará - UFC; 2 Master’s degree, assisting physician, Otorhinolaryngology Unit, HUWC/UFC; 3 Doctoral degree, adjunct professor, Otorhinolaryngology Discipline, Faculdade de Medicina da UFC. Coordinator of the medical residency program in otorhinolaryngology, HUWC/UFC. Otorhinolaryngology Unit, Hospital Universitário Walter Cantídio. Department of Surgery. Universidade Federal do Ceará

**Keywords:** adenoidectomy, quality of life, sleep apnea syndromes

## Abstract

Obstructive Sleep Disorders (OSD) affect mostly the pediatric population. Within this group, its main etiology is adenotonsillar hyperplasia, being adenoidectomy or adenotonsillectomy the best treatment option for quality of life improvement.

**Aim:**

To asses quality of life of children with OSD after adenoidectomy/adenotonsillectomy.

**Method:**

A prospective study was carried out with 48 children, between 2 and 11 years, with clinical manifestations of OSD and obstructive adenotonsillar hyperplasia. The OSA18 questionnaire was answered by the parents to evaluate their children’s quality of life before surgery; at about thirty days and at least eleven months after the procedure. A higher score meant a worse quality of life.

**Results:**

Before surgery, the average OSA18 score was 82.83(SD=12.57), with an average global score for quality of life of 6.04(SD=1.66). Within thirty days after surgery, the average OSA18 score was 34.3 (SD=9.95) with an average global score of 9.6(SD=0.81), both showing significant reduction (p< 0.001). Thirty-four children (70.83%) were re-evaluated between 11 and 30 months (average=16.85; SD=5.16). The average OSA18 score was 35.44 (SD=19.95) with an average global score of 9.28 (SD=1.78). The postoperative evaluations weren’t significantly different.

**Conclusion:**

surgery improves the quality of life of children with OSD, and such improvement maintains for the long run.

## INTRODUCTION

Obstructive sleep disorders (OSDs) consist of a group of diseases characterized by loss of sleep quality resulting in varied clinical repercussions for these patients.

The severity of OSDs in pediatric patients may vary from a mild form - primary snoring - to major conditions such as the upper airway restrictive syndrome (UARS) or the obstructive sleep apnea hypopnea syndrome (OSAHS).

OSDs significantly affect the pediatric population. It is estimated that up to 3% of children have OSAHS,^1^ and that a higher percentage has UARS.^2^ OSDs cause a variety of physical conditions and affect the child’s quality of life, leading to low weight gain, altered cranio-facial development,^3^, ^4^, ^5^ cardiopulmonary sequelae,^3^, ^4^ behavioral changes,^3^, ^4^, ^6^, ^7^ and poor school performance,^8^ all of which have been well documented. Thus an early diagnosis and treatment of OSDs in pediatric patients is paramount to revert or avoid these conditions.

The diagnosis of OSDs is made based on a careful clinical history for which observations parents make about the sleep pattern of their children provide important clues. However, clinical findings alone cannot precisely define if the OSD is a mild condition of mild snoring or the more severe UARS or OSAHS.^9^, ^10^, ^11^ A precise differentiation requires a polysomnographic test, which is the gold standard in such cases.

The literature shows that adenoid or adenotonsillary hyperplasia is the main cause of OSDs in children.^12^, ^13^, ^14^, ^15^ Thus adenoidectomy or adenotonsillectomy are the procedures of choice for treating OSDs in the pediatric population; these procedures effectively control nocturnal symptoms, relieving physical repercussions and normalizing the polysomnographic test.^16^, ^17^, ^18^, ^19^

Although the objective improvement of this condition has been well documented, there have been few reports about subjective improvements in the quality of life of children and the degree of satisfaction of their parents following adenotonsillectomy; the subjective improvement remains presumed.

Recent papers have suggested that children with OSDs have an improved quality of life immediately following surgery, and that their parents report subjectively that the quality of life of their children has improved.^20^, ^21^, ^22^, ^23^, ^24^, ^25^ There remains, however, a gap concerning the long term results about the quality of life of patients undergoing adenotonsillectomy for the treatment of OSDs.

The purpose of this paper was to assess whether well demonstrated short-term quality of life improvements seen in children with OSDs undergoing adenotonsillectomy remain satisfactory in the long-term follow-up of these patients.

## SERIES AND METHOD

We conducted a prospective study of a cohort of 48 children with OSDs and obstructive adenotonsillary hyperplasia. Patients were selected consecutively among those children undergoing adenoidectomy or adenotonsillectomy at the Otorhinolaryngology Unit of the Hospital Universitário Walter Cantídio (HUWC), Universidade Federal do Ceará (UFC), between August 2004 and July 2005.

Prior to surgery all children had complaints suggesting OSD in their clinical history and adenotonsillary hyperplasia documented by the physical examination and nasofibroscopy. Children with comorbidities such as immune deficiencies or craniofacial malformation and children that had been operated previously were excluded.

The caretakers of all children included in this study signed a free informed consent form. The Research Ethics Committee of the HUWC/UFC approved the study (protocol number 105/2004).

All patients were operated under general anesthesia and orotracheal intubation, according to the conventional surgical technique.

Based on the definition of OSDs, children with symptoms and signs that compromised their quality of sleep were included in the study. Additionally, these children had to present obstructive adenotonsillary hyperplasia for which surgery was indicated.

Palatine tonsillary hyperplasia grade III or IV (Brodsky^26^) and/or adenoid hyperplasia that took up at least 70% of the choanae upon deep breathing during nasofibroscopy were considered obstructive hyperplasia.

**Figure 1:**
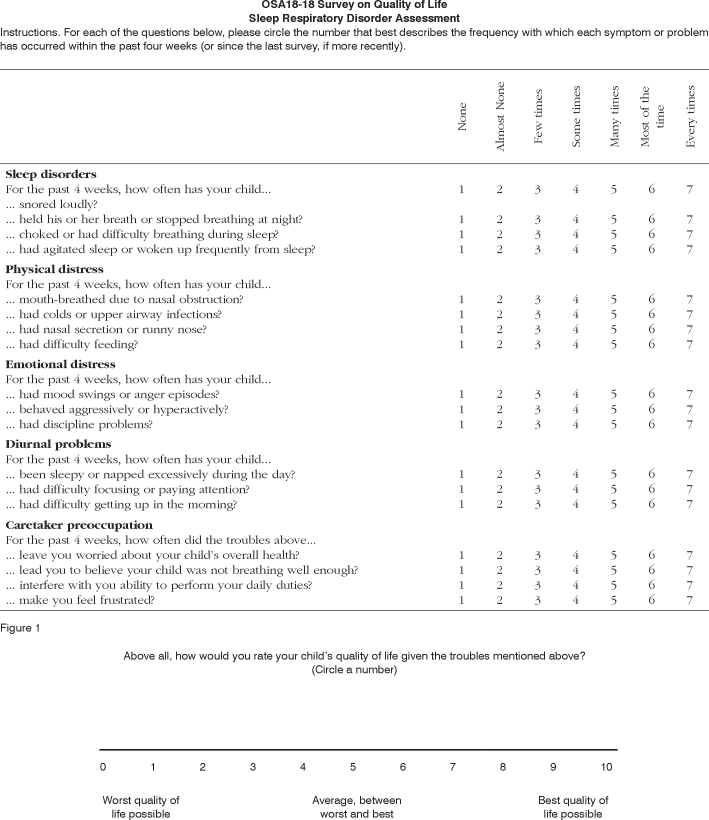
OSA18

The OSA18 questionnaire, developed by Franco Jr. et al.20 and adapted into Portuguese language by Silva et al.,^27^ was applied in three moments to the caretakers of children included in this study to assess the qualify of life of these patients. In the first of these moments - a preoperative visit about 30 days before surgery - a baseline score was obtained as a parameter for assessing the quality of life of children before adenoidectomy or adenotonsillectomy. The questionnaire consists of 18 items grouped into five domains, with questions about sleep disorders, the child’s physical and emotional pain, daily issues for these patients, and the degree of parental concern (Fig. 1).

Caretakers were interviewed and scored from 1 to 7 according to the frequency with which problems defined by each question affected the children. Higher end OSA18 scores correlate with more frequent and important clinical repercussions in the quality of life of children.

OSA18 values may range from 18 to 126 points. According to Franco Jr. et al.,^20^ patients scoring below 60 experience low impact on their quality of life. Patients scoring between 60 and 79 are moderately affected; if the score is 80 points or over, there is major impact on the quality of life.

OSA18 also has a 0 (zero) to 10 (ten) scale in which caretakers may provide an overall score about the patient’s quality of life; in this scale, zero is the worst possible quality of life and 10 is the best possible quality of life.

About one month after surgery the same caretakers answered the questionnaire again to provide a recent postoperative score of the child’s quality of life. Patients were reexamined and submitted to nasofibroscopy to assess the postoperative state of the palatine and pharyngeal tonsillary beds.

Finally, a late postoperative assessment was made of patients at least 11 months after surgery. The OSA18 questionnaire was applied again to the same initial caretaker, and patients underwent a physical exam and nasofibroscopy; the aim was to assess the long-term effects on the quality of life of children.

The software GraphPad Prism v.4.00, da GraphPad Software, Inc. was used for building the graphs and making the statistical analyses. The analysis of variance (ANOVA) with the Tukey post-hoc testing was used for comparing the mean values of the results of three assessments. The statistically significant p value was p < 0.05.

## RESULTS

The results comparing preoperative and early postoperative data (about 30 days after surgery) have been published by Silva et al.,^27^ and are summarized below.

The cohort consisted of 48 children, of which 27 (56.2%) were female and 21 (43.8%) were male. Patients were aged between 2 years and 9 months and 11 years and 3 months (mean - 6 years and 6 months) at the time of adenoidectomy or adenotonsillectomy.

The mean preoperative choanal obstruction per adenoid tissue value was 73.65% (SD=14.86). The mean OSA18 questionnaire preoperative score was 82.83 (SD=12.57); the mean overall quality of life score was 6.04 (SD=1.66).

Children were reassessed about one month after surgery (mean 38.17 days, SD=10.65); at this point only child had persisting adenoid tissue moderately (60%) occluding the cavum. The other children had remaining adenoid tissue occupying 30% or less of the cavum. The mean postoperative OSA18 score was 34.3 (SD=9.95). The mean overall quality of life score was 9.6 (SD=0.81).

A comparison shows that there was a statistically significant reduction (p<0.001) in the postoperative mean overall OSA18 score compared to the corresponding preoperative mean.

These patients were monitored to check whether this improvement persisted in the long term. Early follow-up was done of 48 patients, of which 34 (70.83%) were reassessed in the long term; of these patients, 15 (44.12%) were male and 19 (55.88%) were female. The mean late follow-up assessment period was 16.85 months (SD=5.16), ranging from 11 to 30 months.

The mean late follow-up OSA18 score was 35.44 points (SD=19.96), a low impact of the quality of life. The mean overall quality of life score given by caretakers was 9.28 (SD=1.79).

**Chart 1:**
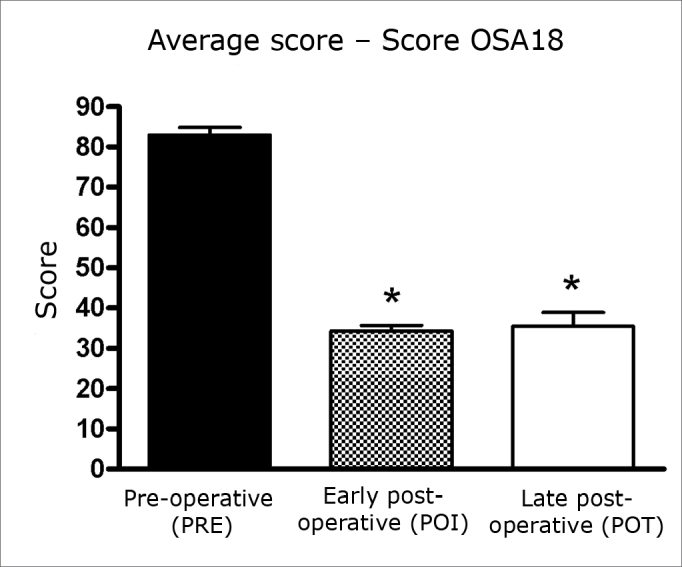
Mean values ± SD of OSA18 scores in three assessments. ANOVA/Tukey: * p < 0.001 (POI vs. PRE; POT vs. PRE).

**Chart 2:**
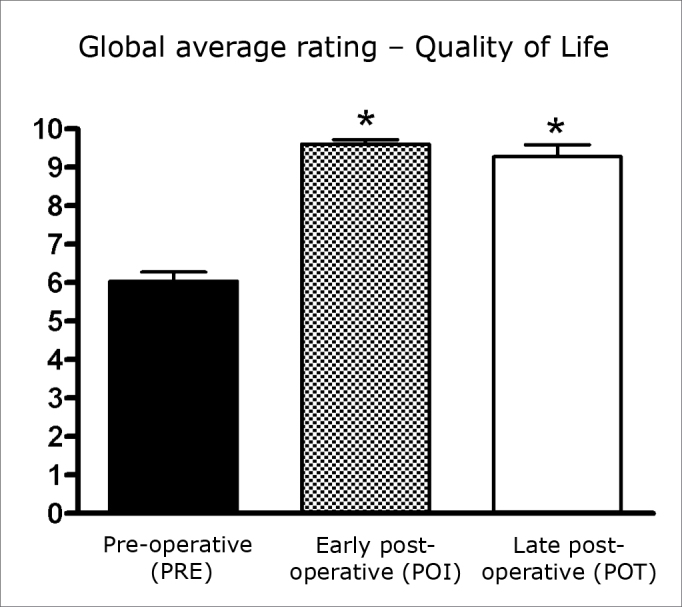
Mean values ± SD of the global score for quality of life in three assessments. ANOVA/Tukey: * p < 0.001 (POI vs. PRE; POT vs. PRE).

In this paper, early postoperative OSA18 scores (34.3; SD=9.95) were compared with late postoperative scores (35.44; SD=19.96). The positive impact on the quality of life was preserved; there was no significant difference between these scores (p>0.05) (Chart 1). There was also no significant difference between the overall scores in these assessments (p>0.05) (Chart 2).

The mean scores in the five questionnaire domains were compared in the three assessment moments. There was a statistically significant reduction (p<0.001) between the preoperative and the early postoperative evaluation in the domains sleep disorders, physical pain and parental concern. The difference was lower, albeit still statistically significant (p<0.05), in the other two domains - emotional pain and daily issues.

**Chart 3:**
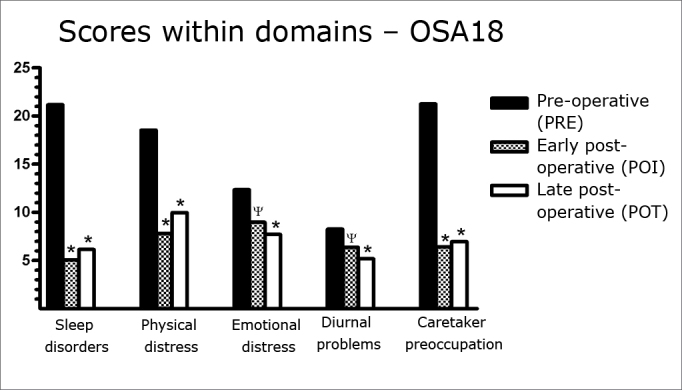
Mean values ± SD of the scores in the five OSA18 questionnaire domains in three assessments. ANOVA/Tukey: * p < 0.001 (POI vs. PRE; POT vs. PRE); ψ p < 0.05 (POI vs. PRE).

There was no statistically significant variation (p>0.05) between the mean early and late postoperative assessments (Chart 3).

Nasofibroscopy was done in 32 of 34 children in the late follow-up review (two children refused to be examined). Adenoid tissue remains filled on average 33.44% of the cavum (SD=20.06).

OSD symptoms recurred in only one of these patients, as seen in the late follow-up visit. This female child had an OSA18 questionnaire score of 75 points in the 14th month after surgery, which was close to her preoperative score (78 points); this corresponds to moderate impact on the quality of life. Nasofibroscopy showed that adenoid tissue filled in 90% of the cavum in this patient, which explains the recurrence of symptoms and poorer quality of life.

One of the patients scored 114 points, with an overall quality of life score equal to zero. Such loss of quality of life was not due to adenoid hyperplasia (nasofibroscopy revealed that adenoid tissue occupied only 30% of the choanae). This patient was diagnosed with allergic rhinitis, for which no treatment was being given, which raised the score.

## DISCUSSION

OSDs are a group of diseases that affect a significant portion of the pediatric population, as shown on epidemiological surveys.^1^, ^2^, ^13^

Current knowledge shows that this group of diseases have negative effects on children in whom an early diagnosis is not made,^3^, ^4^, ^5^, ^6^, ^7^, ^8^ causing major loss of quality of life for the patients and concerns for their caretakers.

Pediatric ENT specialists, therefore, should have knowledge about OSDs to diagnose these conditions early and to provide specific therapy before sequelae develop.

It is a consensus among various authors that adenoid or adenotonsillary hyperplasia is the main cause of OSDs among children;^12^, ^13^, ^14^, ^15^ adenoidectomy (or adenotonsillectomy) have been, therefore, procedures of choice for the treatment of most cases of OSDs in the pediatric population. These procedures have shown positive results in reverting the physical effects of OSDs.^16^, ^17^, ^18^, ^19^

Adenoidectomy/adenotonsillectomy not only revert the organic changes caused by OSDs, but also have shown their value in improving the quality of life of children and the perception of this quality by their caretakers. Recent papers are evidence of this important theme.^20^, ^21^, ^22^, ^23^, ^24^, ^25^

Franco Jr. et al.^20^ was one of the pioneers assessing quality of life improvements following adenotonsillectomy in children with OSDs; in the year 2000 he disseminated a specific questionnaire - the OSA18 - which opened the file for other authors to use this method in assessments.

De Serres et al.^22^ also in 200 developed a similar questionnaire - the OSD6 - that is also an effective tool for assessing the quality of life in children undergoing surgery; its advantage is that it may be used for assessing the postoperative progression of these children.

Sohn et al.^25^ in 2003 undertook a comparative study between the OSA18 and the OSD6 questionnaires by assessing 69 children with OSDs before and after adenotonsillectomy. In this study the OSA18 was superior to the OSD6 in the correlation between its scores and polysomnographic findings,^20^ but failed as a method for evaluating the progression of disease or cure. These authors also concluded that the OSA18 could also provide reliable results in follow-up, since it correlated well with polysomnographic findings.

Di Francesco et al.^28^ in 2004 used the OSD6 questionnaire developed by De Serres et al.^22^ in a pioneering Brazilian study of the abovementioned theme; these authors also concluded that the quality of life of children with OSDs improved following adenotonsillectomy.

Up to this point our study was coherent with the aforementioned papers, which showed shot-term improved quality of life in children with OSDs following adenoidectomy or adenotonsillectomy, as underlined by Silva et al.^27^ Doubts remained, however, about long-term quality of life improvements after surgical therapy, which justifies longer term monitoring of these patients.

Flanary et al.^29^ on this theme, based on OSA18 questionnaire data from parents of patients, showed that positive results persisted six months after surgery. This study also showed that although polysomnograhy provides much information about OSDs, it is unable to establish the degree of quality of life improvement following adenotonsillectomy, as perceived by parents.

Mitchel et al.^30^ used polysomnograhy to assess long-term clinical improvement and showed that quality of life remained 9 to 24 months after adenotonsillectomy in children with OSDs.

The subjects of our study were reassessed on average 16.85 months after surgery. The OSA18 questionnaire was applied again; it is an effective and reliable method for evaluating the progression of these patients, as demonstrated in published studies.^25^, ^29^

Results were satisfactory, demonstrating the quality of life persisted in our patients in the long term after surgery, which confirms published results. Continued quality of life improvement was correlated with nasofibroscopic findings showing that the mean choanal obstruction per adenoid tissue was around 33% in our patients; this percentage is considered mild or even within normal limits.

Our results included one patient with an OSA18 score of 114 points in the late evaluation, an outlier value compared to the other patients. This patient had scored 48 in the early postoperative evaluation, showing a significant improvement after surgery; the preoperative score had been 98. This improvement did not persist in the long term; this patient had allergic rhinitis, and had gone without treatment for many months. The result was that the patient became very symptomatic, with significant nasal block, which raised the scores and worsened the quality of life.

We underline that following adenotonsillectomy, children with allergic rhinitis deserve special monitoring, and should have their allergic symptoms treated to avoid future loss of quality of life.

A possible caveat of this study is the absence of polysomnographic assessments of patients. However, since the aim was to assess quality of life, the OSA18 questionnaire was adequate, since it correlates well with polysomnography.^20^, ^25^ Furthermore, there is not necessarily a correlation between polysomnography and the subjective perception by caretakers of compromised quality of life in these children.^29^

Another possible issue is to apply the OSA18 questionnaire for assessing quality of life I caretakers rather than in the patients themselves. Stewart et al.^21^ has explained the rationale of this approach, suggesting that language complexity and vocabulary differences between the children and researchers make assessing caretakers the best evaluation method for looking at qualify of life in these children.

Pal^31^ supports this rationale by stating that since adults effectively evaluate quality of life losses in children, and are the ones seeking treatment for these children, it is appropriate to apply such a questionnaire to caretakers rather than the children themselves.

## CONCLUSION

Our data show that adenoidectomy/adenotonsillectomy were effective in the treatment of children with OSDs secondary to adenotonsillary hyperplasia, significantly improving their quality of life in the short and long term postoperatively.

We confirmed the validity of the OSA18 questionnaire as a useful tool for assessing the quality of life of children with OSDs before and after adenoidectomy/ adenotonsillectomy.
